# Isolation, genomic analysis and functional characterization of *Enterococcus rotai* CMTB-CA6, a putative probiotic strain isolated from a medicinal plant *Centella asiatica*

**DOI:** 10.3389/fmicb.2024.1452127

**Published:** 2024-09-09

**Authors:** Yunsik Kim, Jin Hee Lee, Jimyeong Ha, Eun-Gyung Cho

**Affiliations:** ^1^Consumer Health 2 Center, CHA Advanced Research Institute, Bundang CHA Medical Center, Seongnam, Republic of Korea; ^2^Consumer Health 1 Center, CHA Advanced Research Institute, Bundang CHA Medical Center, Seongnam, Republic of Korea; ^3^H&B Science Center, CHA Meditech Co., Ltd., Seongnam, Republic of Korea; ^4^Department of Life Science, General Graduate School, CHA University, Pocheon, Republic of Korea

**Keywords:** plant probiotics, lactic acid bacteria, *Enterococcus rotai*, *Centella asiatica*, whole genome sequencing, skin microbiome, anti-inflammation, antioxidant

## Abstract

Probiotics and their derivatives offer significant health benefits by supporting digestive health, boosting the immune system, and regulating the microbiomes not only of the internal gastrointestinal track but also of the skin. To be effective, probiotics and their derivatives must exhibit robust antimicrobial activity, resilience to adverse conditions, and colonization capabilities in host tissues. As an alternative to animal-derived probiotics, plant-derived lactic acid bacteria (LAB) present promising advantages, including enhanced diversity and tolerance to challenging environments. Our study focuses on exploring the potential of plant-derived LAB, particularly from the medicinal plant *Centella asiatica*, in improving skin conditions. Through a bacterial isolation procedure from *C. asiatica* leaves, *Enterococcus rotai* CMTB-CA6 was identified via 16S rRNA sequencing, whole genome sequencing, and bioinformatic analyses. Based on genomic analysis, antimicrobial-resistance and virulence genes were not detected. Additionally, the potential functions of *E. rotai* CMTB-CA6 were characterized by its lysates’ ability to regulate skin microbes, such as stimulating the growth of *Staphylococcus epidermidis* while inhibiting that of *Cutibacterium acnes*, to restore the viability of human dermal fibroblasts under inflammatory conditions, and to demonstrate effective antioxidant activities both in a cell-free system and in human dermal fibroblasts. Our investigation revealed the efficacy of *E. rotai* CMTB-CA6 lysates in improving skin conditions, suggesting its potential use as a probiotic-derived agent for skin care products. Considering the ecological relationship between plant-inhabited bacteria and their host plants, we suggest that the utilization of *E. rotai* CMTB-CA6 strain for fermenting its host plant, *C. asiatica*, could be a novel approach to efficiently enriching bioactive molecules for human health benefits.

## 1 Introduction

Probiotics, primarily composed of lactic acid bacteria (LAB) such as *Lactobacillus*, *Bifidobacterium*, *Enterococcus*, *Bacillus*, and *Pediococcus*, along with several yeasts, confer health benefits on the host (Fijan, 2014; Hill et al., 2014; Zukiewicz-Sobczak et al., 2014). These benefits include supporting a healthy digestive tract and immune system (Kechagia et al., 2013; Fijan, 2014; Hill et al., 2014; Mazziotta et al., 2023). Moreover, probiotics demonstrate effectiveness in regulating the microbiome and facilitating wound healing in both gut and skin tissues through their antimicrobial, anti-inflammatory, and antioxidant properties (Ou et al., 2009; Brandi et al., 2020; Lebeer et al., 2022). For probiotics to be considered effective, they must not only positively regulate the host immune system and exhibit potent antimicrobial activity but also show resilience to acidic environments, successful colonization in host tissues, and prevention of pathogen colonization (Kechagia et al., 2013). To address certain limitations associated with animal-derived probiotics, such as limited diversity and low tolerance to nutrient-depleted, stressful, acidic, and bile environments, LAB sourced from plants are being explored as an alternative. Given the primary emphasis of probiotics on regulating the microbiome in the human intestinal tract, the skin, being another prominent microbial habitat, offers a promising avenue for the application of plant-derived probiotics and related materials. This is particularly relevant considering that the skin serves as a primary target organ for the application of plant-derived materials as cosmeceutical ingredients.

LAB play a vital role in the food industry through their ability to ferment carbohydrates, leading to the production of lactic acid, and in human health as probiotics. These bacteria can originate from various sources, including the human body, plants, soils, and fermented foods. They are typically categorized into animal-derived and plant-derived probiotics. While animal-derived probiotics are generally classified into approximately 12 genera, plant-derived probiotics offer a much broader diversity, encompassing approximately 200 genera (Cho et al., 2009; Yu et al., 2020). Plant-derived probiotics demonstrate enhanced survival rates in challenging environments, such as high salinity and rapid fluctuations in temperature and humidity, and exhibit greater resistance to artificial gastric juices and bile compared to animal-derived probiotics, with survival rates exceeding 90% (Cho et al., 2009; Lamont et al., 2017). Furthermore, plants and probiotics produce a wider range of antimicrobial and physiologically active compounds, including secondary metabolites (Teneva and Denev, 2023). Considering these advantages, plant-derived LAB have been actively isolated and identified from fruits, vegetables, flowers, and medicinal plants, and functionally evaluated through *in vitro*, *ex vivo*, or clinical studies, revealing the beneficial effects on human (Kim et al., 2020; Noda et al., 2021; Danshiitsoodol et al., 2022; Shakya et al., 2022; Shakya et al., 2023).

*Centella asiatica*, a traditional medicinal plant, is well-known for its efficacy in treating various skin issues such as acne, ulcers, eczema, psoriasis, as well as acute and chronic wounds (Diniz et al., 2023). Extensive research has revealed that its active compounds, pentacyclic triterpene glycosides including asiaticoside and madecassoside, along with their respective aglycone moieties, asiatic acid and madecassic acid, offer a broad spectrum of therapeutic properties, including tissue protection, anti-inflammatory, antibacterial, anti-tumor, and immunomodulatory effects (Lv et al., 2018; Biswas et al., 2021; Buranasudja et al., 2021; Bandopadhyay et al., 2023; He et al., 2023). As illustrated by *C. asiatica*, medicinal plants serve as crucial repositories of bioactive molecules, including antibiotics and antivirals. Some of these compounds originate from plant microbiota rather than the plants themselves, highlighting the importance of plant-associated microbiota not only in maintaining plant health but also in influencing therapeutic outcomes (Sen and Samanta, 2015; Castronovo et al., 2021).

Recognizing the potential of plant-associated microbiota as probiotics and their role in offering biotechnological solutions for health issues, we isolated bacterial strains from *C. asiatica* leaves in this study. Subsequently, using 16S rRNA sequencing and whole genome sequencing, we identified one strain, *Enterococcus rotai* CMTB-CA6 and performed functional characterization by assessing the efficacy of bacterial lysates on human skin cells. Our findings suggest that *E. rotai* CMTB-CA6 and its derivatives could be valuable probiotic substances for improving skin conditions. Furthermore, as a LAB, this strain could be directly utilized to ferment *C. asiatica*, a host plant inhabited and associated with *E. rotai* CMTB-CA6, thus effectively increasing the levels of important bioactive molecules for human health benefits.

## 2 Materials and methods

### 2.1 Isolation of a LAB strain CMTB-CA6

The isolation of a LAB strain CMTB-CA6 from *C. asiatica* (Centella), which were collected from nature in Pohang, South Korea, was performed as follows. Briefly, Centella leaves (1 g) were homogenized in phosphate-buffered saline (PBS; Welgene, Korea) and plated on the de Man, Rogosa, and Sharpe (MRS; BD Difco, United States) agar plates for 48 h at 37°C. The morphologically discrete colonies were further subcultured onto MRS agar plates three times to ensure the presence of a clear single colony.

### 2.2 Phylogenetic and comparative analyses

A bacterial isolate CMTB-CA6 was identified morphologically and biochemically, including optical microscopic observation and sugar fermentation ability tests (API 50CHE and API 20E kit, Biomerieux, Frence). The classification of the species and strain of a bacterial isolate CMTB-CA6 was determined by 16S rRNA nucleotide sequence analysis (GenBank accession number PQ084692.1). PCR amplification of the 16S rRNA gene was performed using the universal bacterial primer (27F/1492R; 5′-AGAGTTTGATCCTGGCTCAG-3′/5′-TACGGCTACCTTGTTACGACTT-3′). Species identification was confirmed by comparing the 16S rRNA sequences with related reference strains using the web-based software program BLASTN of GenBank from the national center for biotechnology information (NCBI; https://blast.ncbi.nlm.nih.gov/). Additionally, a phylogenetic tree analysis was performed using the neighbor-joining method based on ClustalW alignment and bootstrapping for further validation.

### 2.3 Whole genome sequencing and *de novo* assembly and bioinformatics analysis

Whole genome sequencing was performed using the SMRT Sequencing system (Pacific Biosciences, United States). Genomic DNA (15 μg/mL) from a bacterial isolate CMTB-CA6 was extracted using the MagAttract HMW DNA Kit (Qiagen, United States), size-selected for 15 kb or longer DNA fragments using Covaris g-TUBE (Covaris, United States), and purified by 0.45X AMPure XP magnetic beads (Beckman Coulter, United States). The DNA library was prepared using the SMRTbell^®^ Express Template Prep Kit 2.0 (PacBio, United States). The DNA Prep Enzyme was used to remove the single-stranded DNA from the ends by incubating the fragmented DNA at 37°C for 15 min. The DNA damage repair mix was applied at 37°C for 30 min to proceed with DNA damage repair process. Subsequently, the End Repair Mix was used at 20°C for 10 min and 65°C for 30 min for the end repair process, followed by the addition of adapters (overhang SMRTbell adapters). Adapter ligation was performed at 20°C for 60 min and 65°C for 10 min to attach the overhang adapters to both ends of the DNA. The DNA was then purified again using 0.45X AMPure XP magnetic beads, and the DNA quantity and size were measured using the ND-2000 spectrophotometer (Thermo Scientific, United States), Qubit^®^ 2.0 Fluorometer (Invitrogen, United States), and Agilent Femto Pulse system (Agilent, United States). Through gel electrophoresis, DNA libraries with sizes of 9–13 kb and 15 kb or more were collected. DNA was recovered using 0.5X AMPure XP magnetic beads, and the size and density of the libraries were quantified. The whole reads from PacBio sequencing were subjected to *de novo* assembly using Flye (v2.8.3). The circular form of the *Enterococcus* genome was confirmed using Circlator (v1.5.5), and the *dnaA* gene was set as the starting point of the genome. This process resulted in a genome sequence coverage of ∼478.7 × for *E. rotai* CMTB-CA6. General features of the genome were organized using Assemblathon (v1.0.0). Cluster of orthologous groups (COG), Gene Ontology (GO), and Kyoto Encyclopedia of Genes and Genomics (KEGG) pathway analysis were performed using Omicsbox (v2.2.4).

### 2.4 Preparation of bacterial lysate and supernatant

*E. rotai* CMTB-CA6 was cultured in 10 mL of MRS medium at 37°C with shaking at 200 rpm for 18–20 h. The optical density (OD_600_) was measured, and 100 μL of the culture was transferred to 10 mL of fresh MRS medium for secondary inoculation. This process was repeated for tertiary inoculation. After culturing, cells were harvested by centrifugation at 3500 rpm for 15 min at 4°C, washed twice with PBS (Welgene, Korea), and resuspended in PBS to a 10% (w/v) concentration. The cells were lysed by sonication using a Branson Ultrasonics Sonifier 450 (Emerson, United States) (sonication conditions: Duration time_1 min 30 s, Duty cycle (pulse)_30%, Output 6; total 7 times) and filtered via a 0.22 μm filter (Merck Millpore, United States) to obtain the whole lysate (WL). The supernatant (Sup) was prepared by centrifuging the WL at 13,000 rpm for 10 min at 4°C. Total protein concentrations in the WL and the Sup were quantified using the Pierce BCA Protein Assay Kit (Thermo Fisher Scientific, United States).

### 2.5 Assessment of the growth-regulating function of skin microbiota

To investigate the interaction between probiotics and skin commensal bacteria, a disk diffusion susceptibility test was conducted (Bauer et al., 1959). *Cutibacterium acnes* was inoculated into actinomyces broth (AB; Mbcell, Korea) and anaerobically cultured at 37°C with shaking at 200 rpm for 18–20 h using an anaerobic pack (Mitsubishi, Japan). The 2.5 mL of *C. acnes* was inoculated into 50 mL of AB medium and incubated for an additional 36 h. The cultured strain was centrifuged at 3,000 rpm for 20 min at 4°C, and the pellet was resuspended in 500 μL of sterile distilled water. A reinforced clostridial medium (1.2% agar; Mbcell, Korea) was prepared at a temperature below 55°C, and the resuspended bacterial solution was added and mixed well before the medium solidified. After approximately 5 h of agar drying, paper discs were placed on the surface. The *E. rotai* CMTB-CA6 lysates and supernatants were individually dispensed onto the paper discs at 15 μL each. The plates were anaerobically cultured at 37°C, and the zone of inhibition was observed. For *Staphylococcus epidermidis*, we conducted the following experiment to investigate the growth-promoting effect of the lysate and supernatant of *E. rotai* CMTB-CA6. *S. epidermidis* was inoculated into nutrient broth (NB; BD Difco, United States) and cultured at 37°C with shaking at 200 rpm for 18–20 h. The first culture was inoculated into 19 mL of NB, and after 9 h of secondary culture, the OD_600_ was measured and adjusted to the range of 0.08–0.12 in a 96-well plate. The probiotic lysate and supernatant (each at 500 μg/mL) were treated at a final concentration of 50 μg/mL (total added volume: 15 μL in 150 μL of NB). After 24 h of incubation at 37°C, the growth of *S. epidermidis* was measured by the OD_600_ to observe whether increased due to the probiotic lysate and supernatant.

### 2.6 Cell viability assay

Human dermal fibroblasts (HDF; ATCC, United States) were cultured in a Dulbecco’s Modified Eagle Medium (DMEM; Welgene, Korea) medium containing 10% fetal bovine serum (FBS; Gibco, United States) and 1% penicillin/streptomycin (Gibco, United States) at 37°C with 5% CO_2_ in an incubator. To assess the cytotoxicity of the probiotic lysate on HDF cells, HDF cells were seeded at a density of 1 × 10^4^ cells per well in a 48-well plate, and the probiotic lysate was treated at various concentrations the next day. After 24 h, 20 μL of EZ-Cytox (DoGenBio, Korea) was added to each well, and absorbance was measured at 450 nm after a 2-h incubation at 37°C with 5% CO_2_. Cytotoxicity was evaluated by confirming if it exceeded 20%. To investigate the anti-inflammatory efficacy of the probiotic lysate on HDF cells treated with TNF-α, HDF cells were seeded at a density of 1 × 10^4^ cells per well in a 48-well plate, and the next day, the medium was replaced with serum-free DMEM, and the cells were serum-starvation for 6 h. Subsequently, TNF-α (20 ng/mL; Sigma-Aldrich, USA) was treated. After 24 h, the probiotic lysate was treated at various concentrations along with 20 ng/mL TNF-α, using FBS as a positive control. After 24 h, EZ-Cytox was added, and absorbance was measured at 450 nm after a 2-h reaction. The effect of the probiotic lysate on recovery of TNF-α-induced cytotoxicity was examined.

### 2.7 Analysis of antioxidant activity

The antioxidant capacity of probiotic extracellular vesicles was assessed using the oxygen radical absorbance capacity (ORAC) method (Kim, 2016). A mixture of 80 nM sodium fluorescein (Sigma-Aldrich, United States) and 125 μL in 75 mM phosphate buffer (pH 7.4) was combined with varying dilutions of probiotic lysate, or vitamin C (25 μL each, Sigma-Aldrich, USA). Subsequently, 50 μL of 75 mM AAPH (2,2′-Azobis(2-methylpropionamidine) dihydrochloride; Sigma-Aldrich, USA) was added, and the reaction proceeded at 37°C for 60 min, with fluorescence measured at excitation 485 nm/emission 528 nm every min. As a negative control, the experiment was conducted by adding only the solvent for sample dilution. The area under the curve (AUC) of the fluorescence decrease curve was calculated using the following mathematical formula: AUC = 1 + *f_1_*/*f*_0_ + … + *f_i_*/*f_0_* + … + *f_60_*/*f_0_* (where *f_0_* is the fluorescence value at 0 min, and *f_i_* represents the fluorescence value at *i* min).

The antioxidant capacity was determined by calculating the net area under the curve (net AUC), obtained by subtracting the AUC value of the negative control from the measured AUC value. For this, probiotic lysate was dissolved in PBS at varying concentrations.

### 2.8 Measurement of intracellular ROS levels

Intracellular ROS levels were determined according to the manufacture’s instruction. In brief, HDFs were treated with WL of *E*. *rotai* CMTB-CA6 for 24 h, followed by treatment with 100 μM of H_2_O_2_ in the presence or absence of WL of *E. rotai* CMTB-CA6 for 1 h. Cells were washed twice with PBS and the DCF fluorescence was measured at excitation and emission wavelengths of 485 and 530 nm, respectively.

### 2.9 Statistical analysis

All data were presented as the means ± standard deviation of at least three independent experiments. Statistical comparisons were carried out by using one-way analysis of variance (ANOVA), followed by Tukey’s *post hoc* test (GraphPad Prism version 8.0.1, GraphPad Software Inc. San Diego, CA, United States). *p* values <0.05 were considered statistically significant.

## 3 Results

### 3.1 Characterization of a bacterial isolate CMTB-CA6 from *Centella asiatica*

**Figure 1:**
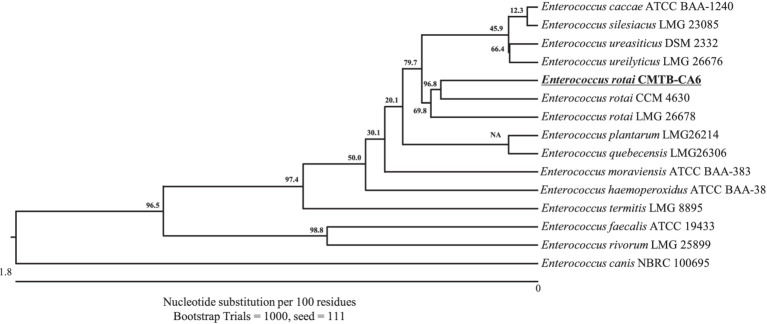
Neighbor-joining phylogenetic tree of bacterial isolate strain CMTB-CA6 with the other *Enterococcus* species. The phylogenetic relationship of *E. rotai* CMTB-CA6 was assessed with the neighbor-joining method of phylogenetics using the 16S rRNA gene sequences of bacterial isolate strain CMTB-CA6 and NCBI database homologs using the ClustalW alignment. Numbers at the node represent bootstrap values in percent for the node (based on 1,000 bootstrap replicates). The GenBank accession number for each 16S rRNA gene sequence are written after relative species name.

A LAB strain CMTB-CA6 was successfully isolated from a medicinal plant *Centella asiatica*. The 16S rRNA sequence (PQ084692.1) of bacterial isolate CMTB-CA6 was searched in the NCBI BLASTN database. Among all other species, *Enterococcus rotai* CCM 4630 (NR_108137.1) and *E. rotai* LMG 26678 (CP013655.1) were ranked at the top of the list with the highest score and shared 99.8% nucleotide identity with a bacterial isolate CMTB-CA6 (Supplementary Table S1) indicating its taxonomic affiliation with *E. rotai*. Phylogenetic analysis was further conducted using the 16S rRNA sequences of *Enterococcus* sp. and showed a bacterial isolate CMTB-CA6 was grouped with *E. rotai* LMG 26678 (the reference sequence of *E. rotai*; CP013655.1) (Figure 1), confirming of its classification into *E. rotai*. According to the analysis of average nucleotide identity (ANI), which represents genetic relatedness between two bacterial genome sequences (Arahal, 2014) and an ANI boundary for taxonomically circumscribing prokaryotic species are approximately 95 ~ 96% (Kim et al., 2019), CMTB-CA6 genomic sequence showed 96.05% of ANI when compared to those of the reference *E. rotai* strains.^1^ Combining the 16S rRNA gene homology, phylogenetic and ANI analyses, a bacterial isolate CMTB-CA6 from *Centella asiatica* is a strain of *E. rotai*, hence labeled as *E. rotai* CMTB-CA6.

Substrate utilization assay of *E. rotai* CMTB-CA6 was performed using both API kits, API 50CHE and API 20E because of no specific kit for *E. rotai* at the time of CMTB-CA6 isolation. Based on these assays, this strain potentially utilizes glycerol, ribose, galactose, D-glucose, D-fructose, D-mannose, N-acetyl glucosamine, amygdaline, arbutine, esculine, salicine, cellobiose, maltose, saccharose, trehalose, melezitose, and β-gentiobiose of the given 49 substrates (API 50CHE) (Supplementary Figures S1A,B). Furthermore, it can activate arginine dihydrolase and produce acetoin during fermentation in addition to using mannitol, inositol, sorbitol, rhamnose, sucrose, melibiose, and arabinose of given substrates as carbon sources (API 20E) (Supplementary Figure S1C).

### 3.2 Genomic features of *Enterococcus rotai* CMTB-CA6

**Figure 2:**
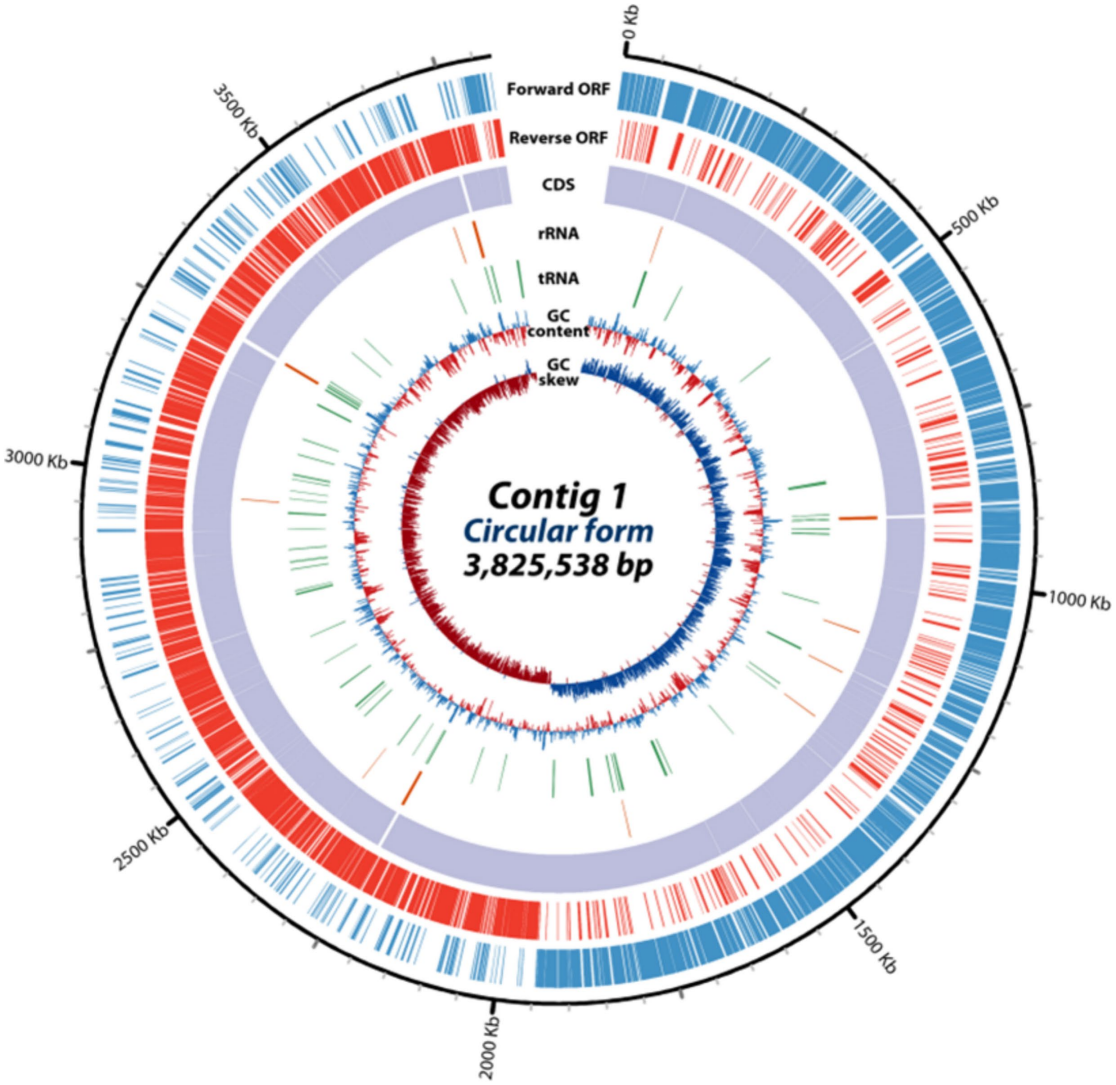
Circular genome map of *E. rotai* CMTB-CA6. The circular genome map was drawn using circulator tool (v1.5.5; https://github.com/sanger-pathogens/circlator) that uses a complete genome sequence and annotates it using Prokka tool (https://github.com/microbial-bioinformatics/prokka). From outside to inside, information is displayed as follows: the predicted ORFs in the forward strand (forward ORF) and reverse strand (reverse ORF), coding sequences (CDS), rRNA genes (red), tRNA genes (green), GC content, GC skew.

Whole-genome sequencing with the PacBio single-molecule real-time (SMRT) sequencing system and *de novo* assembly utilizing the Flye (v2.8.3) and hierarchical genome assembly process 4 (HGAP4) software were employed for the investigation of the genomic features of *E. rotai* CMTB-CA6. The complete genome of *E. rotai* CMTB-CA6 (CP157386.1) was composed of one circular chromosome of a length of 3,825,538 bp with a GC content of 36.48% (Figure 2; Table 1). The sequencing generated a total of 1,831,416,193 subread bases from 179,773 subreads. The N50 of the assembly was 3,825,538, with a total contig length of 3,825,538 bp, and the genome was assembled into a single contig. Among the 3,608 predicted genes, 3,532 genes (≅ 97.89%) were found to be protein-coding sequences (CDSs) and 71 RNA genes (58 tRNA, 12 rRNA, and 1 tmRNA genes; ≅ 1.97%) were identified with no pseudogene (Table 1). Antimicrobial-resistance and virulence genes were not detected in mass screening of contigs using the ABRicate tool (ver. 1.0.1), VirulenceFinder 2.0 (ver. 2.0.5; https://cge.food.dtu.dk/services/VirulenceFinder/), and ResFinder (ver. 4.5.0; http://genepi.food.dtu.dk/resfinder).

**Table 1:** Genome assembly and general features of *Enterococcus rotai* CMTB-CA6 genome.

Attribute	Values
Genome size (bp)	3,825,538
GC content (%)	36.48
Total genes	3,608
CDS (protein)	3,532
Pseudogenes	0
tRNA genes	58
rRNA genes	12
ncRNA genes	0
tmRNA	1

CDS, coding sequence; tRNA, transfer RNA; rRNA, ribosomal RNA; ncRNA, non-coding RNA; tmRNA, transfer-messenger RNA.

### 3.3 Functional classification of protein-coding sequences of *Enterococcus rotai* CMTB-CA6

**Figure 3:**
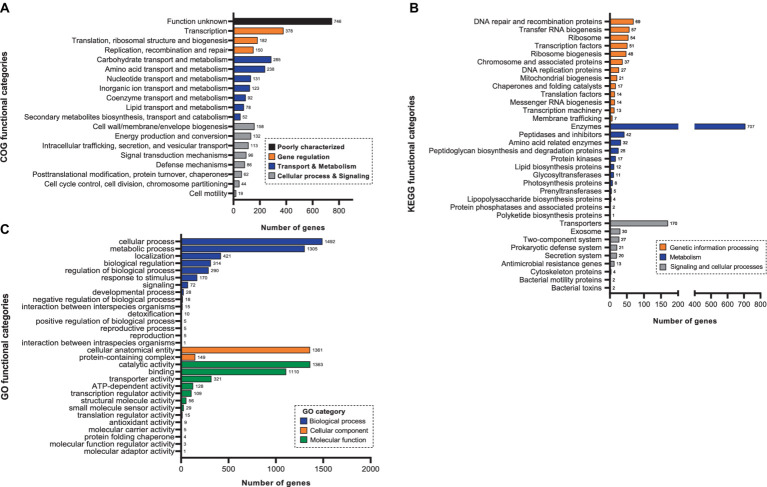
The number of *E. rotai* CMTB-CA6 genes assigned to functional categories. Predicted proteins based on the CDS were assigned to COG, GO and KEGG functional categories with a bioinformatics software Omicsbox (https://www.biobam.com/omicsbox/). **(A)** Clusters of orthologous groups (COG) enrichment analysis. Different bar colors represent the further classification of all functional categories into four major classes: poorly characterized (black bar), gene regulation (orange bars), transport & metabolism (blue bars), cellular processes and signaling (gray bars). **(B)** Gene ontology (GO) enrichment analysis. Different bar colors represent the further classification of all functional categories into three major categories: biological process (blue bars), cellular component (orange bars), molecular function (green bars). **(C)** Kyoto encyclopedia of genes and genomes (KEGG) enrichment analysis. Different bar colors represent the further classification of all functional categories into three major categories: genetic information processing (orange bars), metabolism (blue bars), signaling and cellular processes (gray bars). Among the total 3,532 CDS, 3149 (89.16%), 2,719 (76.98%), and 1984 (56.17%) genes were used for COG, GO, and KEGG analyses, respectively.

Functional classification for 3,532 CDSs of *E. rotai* CMTB-CA6 were performed in three different ways, clusters of orthologous groups (COG), gene ontology (GO), and kyoto encyclopedia of genes and genomes (KEGG) analyses (Figure 3). The COG database is a valuable tool for functional characteristics of newly sequenced microbial genomes and the KEGG analysis is used to examine the diversity, as well as, the functionality of the proteins based on their interactions within biological pathways (Tatusov et al., 2003; Sato et al., 2023). The majority (3,149 CDSs; 89.16%) of 3,532 CDSs were allocated to 19 COG functional categories, showing that “Function unknown” was the most abundant (746 genes, 23.6%), followed by “Transcription” (378 genes, 11.9%), “Carbohydrate transport and metabolism” (285 genes, 9.0%), “Amino acid transport and metabolism” (238 genes, 7.5%), “Translation, ribosomal structure and biogenesis” (182 genes, 5.8%) (Figure 3A). Within these gene groups, those related to transport, metabolism, and biogenesis were 1,452 genes, accounting for 41.11% of the genes allocated to COG functional categories. In the GO analysis, 2,719 CDSs (76.98%) were classified into three different categories, biological process (BP), molecular functions (MF), or cellular component (CC) (Figure 3B). Approximately half of the CDSs (47.10%) were assigned to “Biological process” including cellular process, metabolic process, localization, biological regulation, regulation of biological process, response to stimulus, signaling, developmental process. Secondarily, one third of the CDSs (35.77%) were allocated to “Molecular functions” involving in catalytic activity, binding, transporter activity, ATP-dependent activity, transcription regulator activity, structural molecule activity, small molecule sensor activity (Figure 3B). When KEGG analysis was conducted using 1,984 CDSs (56.17%), the CDSs were assigned to 34 KEGG functional categories and among them, the most abundant category was “Enzymes” (35.6%), followed by transporters, DNA repair and recombination proteins, transcription factors, ribosome, ribosome biogenesis, and transfer RNA biogenesis in order (Figure 3C). Taken together, three distinct protein functional analyses suggest that the majority of CDSs in the *E. rotai* CMTB-CA6 genome are linked to metabolism, transport, and transcription.

### 3.4 Regulatory effect of *Enterococcus rotai* CMTB-CA6 lysates on skin microbes

**Figure 4:**
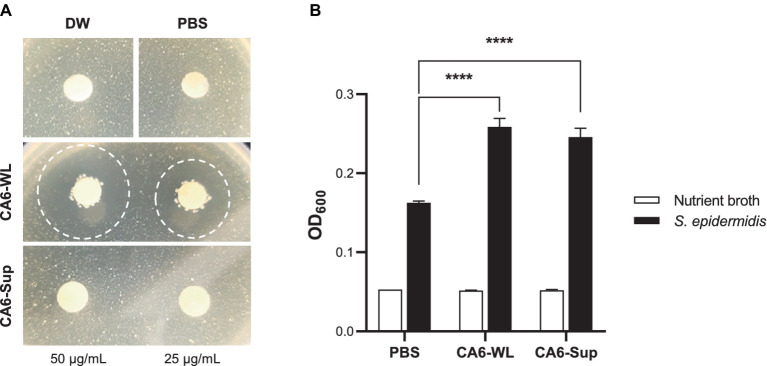
Regulatory effect of *E. rotai* CMTB-CA6 lysates on skin microbes. **(A)** On agar plates containing *Cutibacterium acnes*, paper discs loaded with two different concentrations (25, 50 μg/mL) of whole lysates (WL) and supernatants (Sup) from *E. rotai* CMTB-CA6, or with distilled water (DW) or PBS, were placed (total loaded volume, 15 μL). The agar plates were incubated for 72 h at 37°C. Clear zones formed after loading paper discs were marked with dotted circles. **(B)** In nutrient broth containing *Staphylococcus epidermidis* (150 μL), WL or Sup (each at 500 μg/mL; total added volume, 15 μL) from *E. rotai* CMTB-CA6 were added. After 24 h of culture, the absorbance at 600 nm was measured using a microplate reader. The data are shown as the mean ± standard deviation (*n* = 3; two-way analysis of variance). **** *p* < 0.0001.

*E. rotai* CMTB-CA6 was isolated from *Centella asiatica*, known for its skin wound healing properties and antimicrobial effects (Arribas-Lopez et al., 2022; Diniz et al., 2023). In addition, *Enterococcus* is recognized to have antimicrobial effects as a probiotic (Pieniz et al., 2015), therefore, we investigated whether *E. rotai* CMTB-CA6 possesses antimicrobial effects (Figure 4). In human skin, *Cutibacterium acnes*, a commensal skin bacterial species, has been implicated in the skin condition of acne (Mayslich et al., 2021). Whole lysates (WL) and the supernatants (Sup) of *E. rotai* CMTB-CA6 were subjected to the disk diffusion susceptibility test (also known as the Kirby-Bauer test) (Balouiri et al., 2016). When different concentrations of WL and Sup of *E. rotai* CMTB-CA6 were loaded on discs placed on agar plates where *C. acnes* were growing, only WL parts inhibited the growth of *C. acnes* in a dose-dependent manner, producing different size of clear zones of inhibition (Figure 4A). Compared to WL, Sup of *E. rotai* CMTB-CA6, PBS, and distilled water did not produce a zone of inhibition. This result suggests that WL of *E. rotai* CMTB-CA6 can inhibit the growth and colony formation of *C. acnes*.

Because *E. rotai* CMTB-CA6 could inhibit the growth of *C. acnes*, an opportunistic pathogen, we investigate whether it can regulate the growth of another skin commensal bacterium, *Staphylococcus epidermidis*, known to be associated with maintaining a healthy skin microbiome and involved in suppressing skin pathogens, modulating the immune system, and wound repair (Otto, 2009; Landemaine et al., 2023). WL and Sup of *E. rotai* CMTB-CA6 were inoculated into the culture media where *S. epidermidis* were growing, and the bacterial growth was assessed by measuring the optical density (OD_600_) values. Treatment of NB growth media, CA6-WL or CA6-Sup did not cause the change in OD_600_ values in the absence of *S. epidermidis* inoculation (Figure 4B, white bars). Upon inoculation of *S. epidermidis* into NB growth media, OD_600_ at 24 h post-inoculation at 37°C was well-increased, indicating the successful growth of *S. epidermidis* (Figure 4B, 1st black bar). When WL or Sup of *E. rotai* CMTB-CA6 were applied into *S. epidermidis*-inoculated NB growth media, the growth of *S. epidermidis* was further increased than in NB growth media alone (Figure 4B, 2nd and 3rd black bars), suggesting that WL and Sup of *E. rotai* CMTB-CA6 can promote the growth of *S. epidermidis*. Assumed from the results of successful regulation in the growth of two skin commensal bacteria, *C. acnes* and *S. epidermidis*, *E. rotai* CMTB-CA6 may play a role as potential probiotics on human skin by revealing not only antimicrobial activity against pathogens but also growth-promoting activity for beneficial bacteria.

### 3.5 Anti-cytotoxic and anti-inflammatory effects of *Enterococcus rotai* CMTB-CA6 lysates on human dermal fibroblasts

**Figure 5:**
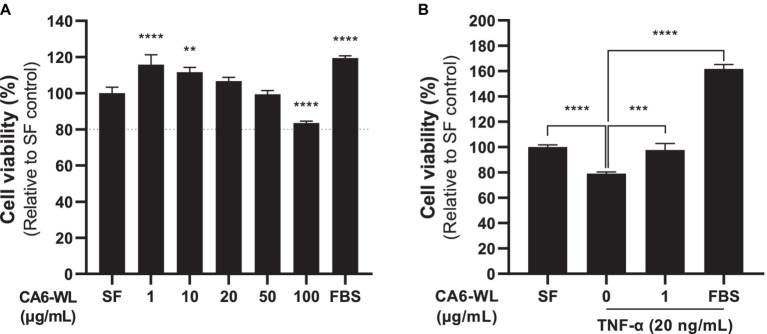
Anti-cytotoxic and anti-inflammatory effects of *E. rotai* CMTB-CA6 lysates on human dermal fibroblasts. **(A)** Human dermal fibroblasts were treated whole lysates (WL) of *E. rotai* CMTB-CA6 (CA6-WL) with various concentrations. Cell viability was assessed using the water-soluble tetrazolium salt (WST) assay. A dotted line indicates 80% of cell viability, signifying cytotoxicity when cell viability falls below this line. **(B)** Human dermal fibroblasts were serum starved for 6 h and then treated with 20 ng/mL of TNF-α for 24 h in serum-free conditions. Various CA6-WL concentrations were treated for an additional 24 h in the presence of TNF-α. Cell viability was determined via the WST assay. As a positive control, 10% FBS was used. The data represent the mean ± standard deviation (*n* = 3; one-way analysis of variance). ** *p* < 0.01, *** *p* < 0.001, **** *p* < 0.0001. TNF, tumor necrosis factor; SF, serum-free control.

Hyperinflammation impacts various skin disease and wound healing (Otto, 2009; Dissemond and Romanelli, 2022). Both *Centella asiatica* and the probiotic *Enterococcus* have been well-known for their anti-inflammatory functions (Goo et al., 2018; Park, 2021; Diniz et al., 2023). We initially examined whether whole lysate of *E. rotai* CMTB-CA6 (CA6-WL) induced cellular toxicity in human dermal fibroblasts (HDFs). Among the tested concentrations, there was no significant increase in cell death. In fact, cells at lower concentrations (20 μg/mL or less) exhibited higher viability (Figure 5A). Consequently, we used 1 μg/mL of CA6-WL for subsequent experiments. To evaluate the potential anti-inflammatory properties of *E. rotai* CMTB-CA6, we treated cells with TNF-α, a major inflammatory cytokine secreted under stressful skin conditions and during aging (Jang et al., 2021; van Loo and Bertrand, 2023) and investigated whether CA6-WL could restore cell viability diminished by TNF-α treatment. TNF-α treatment decreased the cell viability of HDFs compared to a serum-free control, and this effect was reversed by treatment with CA6-WL or 10% fetal bovine serum, a broad-range positive control (Figure 5B). These results suggest that CA6-WL treatment can restore TNF-α-induced inflammatory phenotypes in HDFs.

### 3.6 Antioxidant effect of *Enterococcus rotai* CMTB-CA6 lysates on human dermal fibroblasts

**Figure 6:**
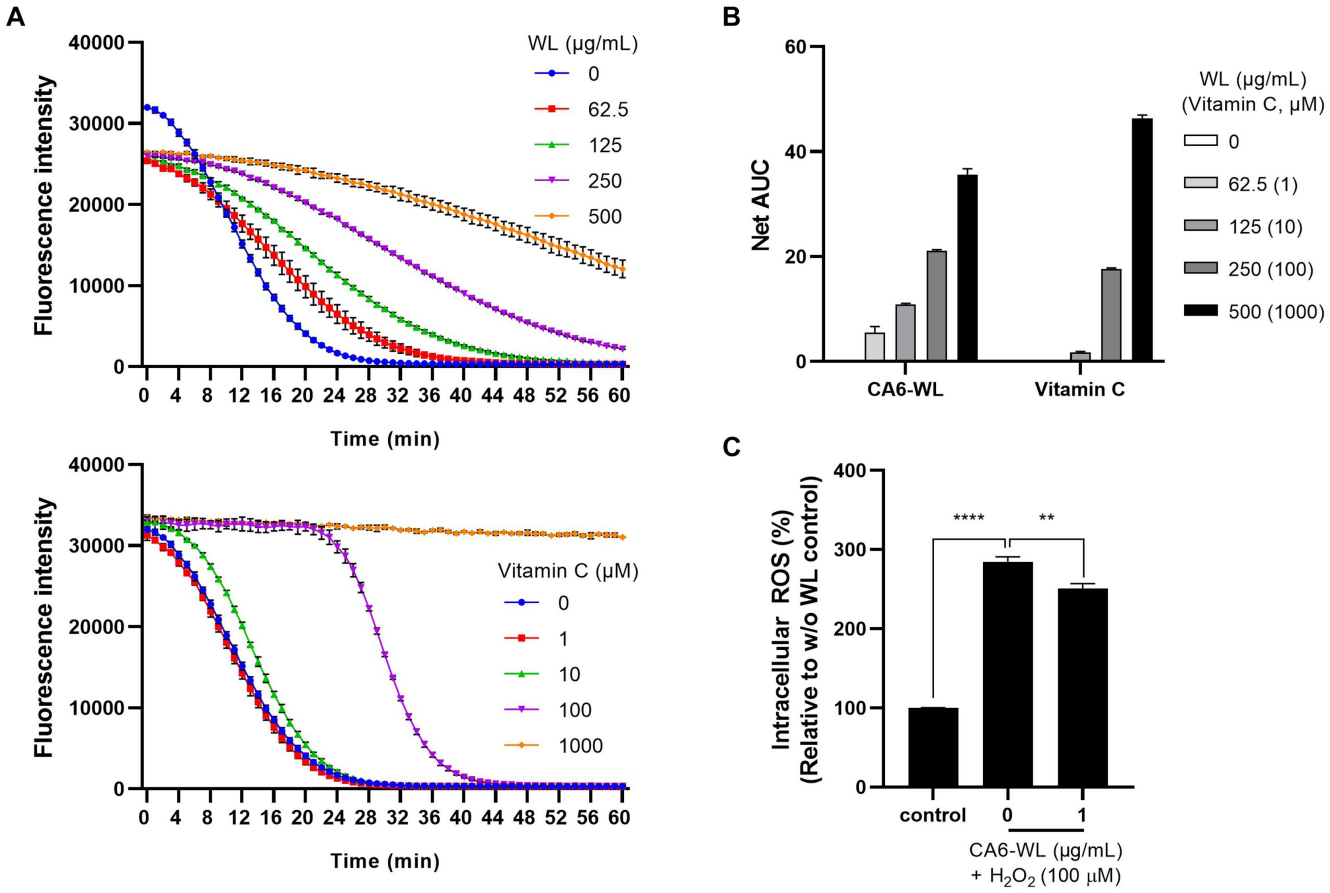
Antioxidant activity of *E. rotai* CMTB-CA6 lysates. **(A)** The antioxidant activities of whole lysates (WL) of *E. rotai* CMTB-CA6 (top) and vitamin C (bottom) at various concentrations were assessed using the oxygen radical absorbance capacity (ORAC) assay. Florescent signals were measured every minute during the 60-min incubation period at 37°C. **(B)** In each group, the net area under the curve (AUC) was calculated by following formula: AUC (sample)-AUC (blank). The data are shown as the mean ± SD (*n* = 3). Vitamin C was used as a positive control. **(C)** Intracellular ROS levels were assessed using CM-H_2_DCFDA, a cellular ROS indicator. HDFs treated with CM-H_2_DCFDA (1 μM) were incubated with WL of *E. rotai* CMTB-CA6 at different concentrations (0, 62.5, 125, 250, 500 μg/mL) in the presence of H_2_O_2_ for 1 h. Fluorescence signals at 530 nm were measured using a fluorometer. Vitamin C was employed as a positive control. The data represent the mean ± standard deviation (*n* = 3; two-way analysis of variance). ** *p* < 0.01, **** *p* < 0.0001.

In addition to its anti-inflammatory properties, the antioxidant potential of probiotic *Enterococcus* has also been suggested (Abdhul et al., 2014; Pieniz et al., 2014a; Pieniz et al., 2014b; Divyashri et al., 2015; Pieniz et al., 2015). To evaluate whether the WL of the probiotic *E. rotai* CMTB-CA6 exhibits antioxidant activity, an oxygen radical absorbance capacity (ORAC) assay was conducted. In the ORAC assay, reactive oxygen species (ROS) generated by AAPH quench fluorescence, leading to a reduction in fluorescence intensity. By adding an antioxidant substance and monitoring this process for 1 h, the ROS-scavenging ability of the antioxidant and its duration can be measured. Various concentrations of CA6-WL or vitamin C, a representative antioxidant, were included in the ORAC reaction, and the fluorescence intensity was measured during the 1-h incubation period. As the concentration of CA6-WL (top panel) or vitamin C (bottom panel) increased, the fluorescence intensity maintained a nearly linear level up to a certain point and then decreased (Figure 6A). The decrease was gradual for CA6-WL but rapid for vitamin C at concentrations of 100 μM or below (Figure 6A). This resulted in gradual but differential increases in the area under the curve (AUC) values for the respective substances (Figure 6B), suggesting that CA6-WL maintains relatively stable antioxidant properties. Besides the cell-free antioxidant assay, we examined whether CA6-WL could lower H_2_O_2_-induced ROS levels in HDFs. Treatment of HDFs with H_2_O_2_ led to an increase in intracellular ROS levels by more than 2.8-fold compared to untreated control cells. However, treatment with CA6-WL at a concentration of 1 μg/mL reversed this effect (Figure 6C). Since excessive exposure to ROS is a major cause of skin inflammation and aging (Arulselvan et al., 2016), and several antioxidants are known to reduce skin inflammation (Fuller, 2022), CA6-WL, with its stable antioxidant capacity, can serve as a potential probiotic-derived substance for mitigating skin inflammation and aging-related phenotypes.

## 4 Discussion

Probiotic LAB, for example, *Lactobacillus* spp. and *Bacillus* spp. are being extensively explored and characterized for their potential use for human health and skin care. Various LAB have been isolated from milk, human gastrointestinal tract (GIT), fermented foods, as well as plants (Shokryazdan et al., 2017; Yadav et al., 2020). Recently, LAB associated with medicinal plants such as from the rhizosphere of the medicinal plants *Ocimum tenuiflorum*, *Azadirachta indica*, *Ficus carica* were massively isolated and some of strains were characterized as potential probiotics showing antioxidant, anti-inflammatory and anti-diabetic effects (Khan et al., 2021). However, there is no information on the role of LAB associated with *Centella asiatica*, a representative medicinal plant, as a substance for human health and skin care. In this study, we isolated and identified *E. rotai* CMTB-CA6 from *Centella asiatica* leaves, not from the plant’s rhizosphere, and characterized it for its potential use as a probiotic strain with the capability to regulate the human skin microbiome, as well as for its antioxidant and anti-inflammatory efficacy.

*Enterococcus* spp., especially *E. faecium* and *E. faecalis*, are traditionally classified as LAB and are commonly found in the GIT of human and farm animals, as well as in animal-derived or dairy foods (Klein, 2003; Fisher and Phillips, 2009). Consistent with their origin, probiotic strains of *Enterococcus* spp. are known to offer beneficial effects in gastrointestinal disorders, by improving acute diarrhea, modulating intestinal microflora, and enhancing immune function (Divyashri et al., 2015). *Enterococcus* spp. have also been studied for their beneficial effects on the skin, such as improving atopic dermatitis, exhibiting antimicrobial activity against *C*. *acnes*, and aiding in the treatment of skin lesions (Kang et al., 2009; Choi et al., 2016). These effects were mainly attributed to antimicrobial, antioxidant, and anti-inflammatory properties (Divyashri et al., 2015; Pieniz et al., 2015; Choi et al., 2018; Hanchi et al., 2018). Although *Enterococcus* spp. are highly competitive due to their resistance to wide range of pH, heat and extreme salinity, and their ability to produce bacteriocins potentially recognized as natural antimicrobial agents in the food industry and as antibiotics candidates, there are possibly safety concerns related to the prevalence of virulence factors and antibiotic-resistance genes and the ability to cause disease (Fisher and Phillips, 2009; Hanchi et al., 2018; Fugaban et al., 2021). Considering their safety concerns, the source of the isolated strains of *Enterococcus* spp. and the absence of virulence and antibiotic-resistance genes are crucial factors for their use as probiotics. Additionally, the probiotic efficacy and safety of new *Enterococcus* spp. strains should be assessed in human intestinal cell lines to demonstrate their potential for adherence and lack of cytotoxicity in the human GIT when administered live via ingestion.

Contrast to other common isolates of *Enterococcus* spp. from animal-derived or dairy foods, *E. rotai* CMTB-CA6 was isolated from the leaves of *Centella asiatica*, a medicinal herbaceous plant. An analysis of the complete genome sequence of *E. rotai* CMTB-CA6 revealed no virulence factors or antibiotic resistance genes. Furthermore, treatment with relatively high concentrations of whole lysates of *E. rotai* CMTB-CA6 (100 μg/mL) showed no cytotoxicity in human dermal fibroblasts (Figure 5A). In addition to its characteristics of lacking virulence factors, antibiotic resistance genes, and cytotoxicity, *E. rotai* CMTB-CA6, which exhibits antioxidant and anti-inflammatory effects on human skin cells, as well as antimicrobial activity against *C. acnes* but not *S. epidermidis*, can be used as a promising probiotic for human health and skin care by helping to regulate the human skin microbiome and improve inflammatory skin conditions. Although our study demonstrated relatively favorable characteristics of *E. rotai* CMTB-CA6 *in vitro* and for use as a cosmetic ingredient, it still needs to be evaluated under *in vivo* conditions to determine its suitability for use in the food industry.

Probiotic strains that reside in the intestinal tract positively affect the human digestive and immune systems (Papadimitriou et al., 2015; Mazziotta et al., 2023). The physiology, including the metabolic processes, of probiotic strains can be influenced by their environment, facilitating the sharing of metabolic byproducts with the host (Rafique et al., 2023). Similarly, the interaction between *C. asiatica* and its microbiota also enables the exchange of metabolic byproducts, which could contribute to *C. asiatica*’s therapeutic efficacy. Whole lysates of *E. rotai* CMTB-CA6, cultured in MRS medium without the original host plant, showed antimicrobial, anti-inflammatory, and antioxidant effects on human skin cells. These effects may be due to the inherent properties of *Enterococcus* spp. or its association with *C. asiatica* as its LAB strain, through which the metabolic system of *E. rotai* CMTB-CA6 has been developed. Therefore, *E. rotai* CMTB-CA6 could be an appropriate LAB strain for fermenting *C. asiatica* when needed.

*Centella asiatica*, a medicinal herbaceous plant widely used in traditional oriental medicine for wound healing, skin diseases, and enhancing brain function (Sun et al., 2020; Diniz et al., 2023). Its bioactive constituents, including asiaticoside, madecassoside, asiatic acid, and madecassic acid, exhibit various pharmacological benefits, such as neuroprotective, wound-healing, and skin-protective properties, leading to their widespread use in a variety of clinical and cosmetic treatments (Goo et al., 2018; Arribas-Lopez et al., 2022; Bandopadhyay et al., 2023; He et al., 2023; Rashid et al., 2023). Despite the various therapeutic effects of *C. asiatica* extract and its active constituents, caution is advised regarding the concentrations and durations of treatment, as some side effects have been reported in a few clinical cases (Sun et al., 2020; Rashid et al., 2023). One method to improve efficacy by enhancing active components or facilitating digestion and absorption while reducing the side effects of raw materials could be to ferment them using LAB or bacterial enzymes, as is commonly done in ginseng fermentation (Majchrzak et al., 2022). Since choosing the right microorganisms is crucial to achieve a desirable mix of biologically active compounds in the fermentation process, *E. rotai* CMTB-CA6, a LAB strain that inhabited and was isolated from *C. asiatica* leaves, could be an excellent choice for fermenting host plant. This could result in fermented raw materials that are enriched with active triterpenes and are more compatible with human tissues compared to unfermented raw materials. With this in mind, we are currently conducting experiments to evaluate the use of *E. rotai* CMTB-CA6 as a probiotic for fermenting *C. asiatica*.

As the largest organ of the human body, the skin hosts millions of bacteria, fungi, and viruses that make up the skin microbiota (Byrd et al., 2018). The skin is colonized by beneficial microorganisms and acts as a physical barrier to prevent pathogen invasion. When the balance between commensals and pathogens is disrupted, it can lead to skin diseases or even systemic diseases (Byrd et al., 2018). Therefore, the ability to regulate the skin microbiota should be considered when developing bacteria-derived materials as cosmetic ingredients. Since live microbes like probiotics are not allowed in cosmetics (Dou et al., 2023), bacterial lysates, ferments, or filtrates could be considered as suitable alternatives.

In our study, whole lysates of *E. rotai* CMTB-CA6 showed potential for regulating the skin microbiome by inhibiting the growth of *C. acnes* and promoting that of *S. epidermidis*. They also exhibited antioxidant and anti-inflammatory properties without causing cellular toxicity. This suggests that *E. rotai* CMTB-CA6 could be effective in enhancing skin barrier function and reducing signs of skin aging, and thus could be used as a potential probiotic strain for human health and skin care.

## Data availability statement

The datasets presented in this study can be found in online repositories. The names of the repository/repositories and accession number(s) can be found at: https://www.ncbi.nlm.nih.gov/genbank/, CP157386.1; https://www.ncbi.nlm.nih.gov/bioproject/, PRJNA1117222; and https://www.ncbi.nlm.nih.gov/genbank/, PQ084692.1.

## Ethics statement

Ethical approval was not required for the studies on humans in accordance with the local legislation and institutional requirements because only commercially available established cell lines were used. Ethical approval was not required for the studies on animals in accordance with the local legislation and institutional requirements because only commercially available established cell lines were used.

## Author contributions

YK: Data curation, Formal analysis, Investigation, Methodology, Resources, Software, Validation, Visualization, Writing – original draft. JL: Data curation, Formal analysis, Investigation, Methodology, Validation, Writing – original draft. JH: Data curation, Formal analysis, Methodology, Validation, Writing – original draft. E-GC: Conceptualization, Data curation, Formal analysis, Funding acquisition, Investigation, Methodology, Project administration, Resources, Supervision, Writing – original draft, Writing – review & editing.

## Footnotes

^1^ http://enve-omics.ce.gatech.edu/ani/

## References

[ref1] Abdhul K. Ganesh M. Shanmughapriya S. Kanagavel M. Anbarasu K. Natarajaseenivasan K. (2014). Antioxidant activity of exopolysaccharide from probiotic strain *Enterococcus faecium* (BDU7) from Ngari. Int. J. Biol. Macromol. 70, 450–454. doi: 10.1016/j.ijbiomac.2014.07.026 25062992

[ref2] Arahal D. R. (2014). “Chapter 6—whole-genome analyses: average nucleotide identity” in Methods in microbiology. eds. Goodfellow M. Sutcliffe I. Chun J. (Amsterdam: Elsevier), 103–122.

[ref3] Arribas-Lopez E. Zand N. Ojo O. Snowden M. J. Kochhar T. (2022). A systematic review of the effect of Centella asiatica on wound healing. Int. J. Environ. Res. Public Health 19:3266. doi: 10.3390/ijerph19063266, PMID: 35328954 PMC8956065

[ref4] Arulselvan P. Fard M. T. Tan W. S. Gothai S. Fakurazi S. Norhaizan M. E.. (2016). Role of antioxidants and natural products in inflammation. Oxidative Med. Cell. Longev. 2016:5276130. doi: 10.1155/2016/5276130, PMID: 27803762 PMC5075620

[ref5] Balouiri M. Sadiki M. Ibnsouda S. K. (2016). Methods for in vitro evaluating antimicrobial activity: a review. J Pharm Anal 6, 71–79. doi: 10.1016/j.jpha.2015.11.005, PMID: 29403965 PMC5762448

[ref6] Bandopadhyay S. Mandal S. Ghorai M. Jha N. K. Kumar M. Radha. (2023). Therapeutic properties and pharmacological activities of asiaticoside and madecassoside: a review. J. Cell. Mol. Med. 27, 593–608. doi: 10.1111/jcmm.17635, PMID: 36756687 PMC9983323

[ref7] Bauer A. W. Perry D. M. Kirby W. M. (1959). Single-disk antibiotic-sensitivity testing of staphylococci; an analysis of technique and results. A.M.A. Arch. Intern. Med. 104, 208–216. doi: 10.1001/archinte.1959.00270080034004, PMID: 13669774

[ref8] Biswas D. Mandal S. Chatterjee Saha S. Tudu C. K. Nandy S. Batiha G. E.-S.. (2021). Ethnobotany, phytochemistry, pharmacology, and toxicity of *Centella asiatica* (L.) urban: a comprehensive review. Phytother. Res. 35, 6624–6654. doi: 10.1002/ptr.7248, PMID: 34463404

[ref9] Brandi J. Cheri S. Manfredi M. Di Carlo C. Vita Vanella V. Federici F.. (2020). Exploring the wound healing, anti-inflammatory, anti-pathogenic and proteomic effects of lactic acid bacteria on keratinocytes. Sci. Rep. 10:11572. doi: 10.1038/s41598-020-68483-4, PMID: 32665600 PMC7360600

[ref10] Buranasudja V. Rani D. Malla A. Kobtrakul K. Vimolmangkang S. (2021). Insights into antioxidant activities and anti-skin-aging potential of callus extract from *Centella asiatica* (L.). Sci. Rep. 11:13459. doi: 10.1038/s41598-021-92958-7, PMID: 34188145 PMC8241881

[ref11] Byrd A. L. Belkaid Y. Segre J. A. (2018). The human skin microbiome. Nat. Rev. Microbiol. 16, 143–155. doi: 10.1038/nrmicro.2017.157, PMID: 29332945

[ref12] Castronovo L. M. Vassallo A. Mengoni A. Miceli E. Bogani P. Firenzuoli F.. (2021). Medicinal plants and their bacterial microbiota: a review on antimicrobial compounds production for plant and human health. Pathogens 10:106. doi: 10.3390/pathogens10020106, PMID: 33498987 PMC7911374

[ref13] Cho Y.-H. Park S.-N. Jeong S.-H. (2009). A study on the physiological activity and industrial prospects of plant-origin lactic acid Bacteria. Dairy Sci. Biotechnol. 27:53.

[ref14] Choi M.-S. Chang S.-J. Chae Y. Lee M.-H. Kim W.-J. Iwasa M.. (2018). Anti-inflammatory effect of heat-killed Enterococcus faecalis, EF-2001. J. Life Sci. 28, 1361–1368. doi: 10.5352/JLS.2018.28.11.1361

[ref15] Choi E. J. Iwasa M. Han K. I. Kim W. J. Tang Y. Hwang Y. J.. (2016). Heat-killed *Enterococcus faecalis* EF-2001 ameliorates atopic dermatitis in a murine model. Nutrients 8:146. doi: 10.3390/nu8030146 26959058 PMC4808875

[ref16] Danshiitsoodol N. Noda M. Kanno K. Uchida T. Sugiyama M. (2022). Plant-derived Lactobacillus paracasei IJH-SONE68 improves the gut microbiota associated with hepatic disorders: a randomized, double-blind, and placebo-controlled clinical trial. Nutrients 14:4492. doi: 10.3390/nu14214492, PMID: 36364756 PMC9657077

[ref17] Diniz L. R. L. Calado L. L. Duarte A. B. S. de Sousa D. P. (2023). Centella asiatica and its metabolite Asiatic acid: wound healing effects and therapeutic potential. Meta 13:276. doi: 10.3390/metabo13020276, PMID: 36837896 PMC9966672

[ref18] Dissemond J. Romanelli M. (2022). Inflammatory skin diseases and wounds. Br. J. Dermatol. 187, 167–177. doi: 10.1111/bjd.21619 35514247

[ref19] Divyashri G. Krishna G. Muralidhara Prapulla S. G. (2015). Probiotic attributes, antioxidant, anti-inflammatory and neuromodulatory effects of *Enterococcus faecium* CFR 3003: in vitro and in vivo evidence. J. Med. Microbiol. 64, 1527–1540. doi: 10.1099/jmm.0.000184 26450608

[ref20] Dou J. Feng N. Guo F. Chen Z. Liang J. Wang T.. (2023). Applications of probiotic constituents in cosmetics. Molecules 28:6765. doi: 10.3390/molecules28196765, PMID: 37836607 PMC10574390

[ref21] Fijan S. (2014). Microorganisms with claimed probiotic properties: an overview of recent literature. Int. J. Environ. Res. Public Health 11, 4745–4767. doi: 10.3390/ijerph110504745, PMID: 24859749 PMC4053917

[ref22] Fisher K. Phillips C. (2009). The ecology, epidemiology and virulence of Enterococcus. Microbiology (Reading) 155, 1749–1757. doi: 10.1099/mic.0.026385-0 19383684

[ref23] Fugaban J. I. I. Holzapfel W. H. Todorov S. D. (2021). Probiotic potential and safety assessment of bacteriocinogenic Enterococcus faecium strains with antibacterial activity against Listeria and vancomycin-resistant enterococci. Curr. Res. Microb. Sci. 2:100070. doi: 10.1016/j.crmicr.2021.100070, PMID: 34841360 PMC8610289

[ref24] Fuller B. B. (2022). “Antioxidants and anti-inflammatories,” in Cosmetic dermatology, ed. Draelos Z. D. (Hoboken, New Jersey: Wiley), 366–387.

[ref25] Goo Y.-M. Kil Y. S. Sin S. M. Lee D. Y. Jeong W. M. Ko K.. (2018). Analysis of antibacterial, anti-inflammatory, and skin-whitening effect of *Centella asiatica* (L.) urban. J. Plant Biotechnol. 45, 117–124. doi: 10.5010/jpb.2018.45.2.117

[ref26] Hanchi H. Mottawea W. Sebei K. Hammami R. (2018). The genus Enterococcus: between probiotic potential and safety concerns-an update. Front. Microbiol. 9:1791. doi: 10.3389/fmicb.2018.01791 30123208 PMC6085487

[ref27] He Z. Hu Y. Niu Z. Zhong K. Liu T. Yang M.. (2023). A review of pharmacokinetic and pharmacological properties of asiaticoside, a major active constituent of *Centella asiatica* (L.) Urb. J. Ethnopharmacol. 302:115865. doi: 10.1016/j.jep.2022.115865 36306932

[ref28] Hill C. Guarner F. Reid G. Gibson G. R. Merenstein D. J. Pot B.. (2014). Expert consensus document. The international scientific Association for Probiotics and Prebiotics consensus statement on the scope and appropriate use of the term probiotic. Nat. Rev. Gastroenterol. Hepatol. 11, 506–514. doi: 10.1038/nrgastro.2014.66 24912386

[ref29] Jang D. I. Lee A. H. Shin H. Y. Song H. R. Park J. H. Kang T. B.. (2021). The role of tumor necrosis factor alpha (TNF-alpha) in autoimmune disease and current TNF-alpha inhibitors in therapeutics. Int. J. Mol. Sci. 22:2719. doi: 10.3390/ijms22052719, PMID: 33800290 PMC7962638

[ref30] Kang B. S. Seo J. G. Lee G. S. Kim J. H. Kim S. Y. Han Y. W.. (2009). Antimicrobial activity of enterocins from *Enterococcus faecalis* SL-5 against *Propionibacterium acnes*, the causative agent in acne vulgaris, and its therapeutic effect. J. Microbiol. 47, 101–109. doi: 10.1007/s12275-008-0179-y, PMID: 19229497

[ref31] Kechagia M. Basoulis D. Konstantopoulou S. Dimitriadi D. Gyftopoulou K. Skarmoutsou N.. (2013). Health benefits of probiotics: a review. ISRN Nutr. 2013:481651. doi: 10.5402/2013/481651, PMID: 24959545 PMC4045285

[ref32] Khan A. N. Yasmin H. Ghazanfar S. Hassan M. N. Keyani R. Khan I.. (2021). Antagonistic, anti-oxidant, anti-inflammatory and anti-diabetic probiotic potential of *Lactobacillus agilis* isolated from the rhizosphere of the medicinal plants. Saudi J. Biol. Sci. 28, 6069–6076. doi: 10.1016/j.sjbs.2021.08.029 34764740 PMC8568817

[ref33] Kim S. J. (2016). Antioxidant activity and inhibitory effect of melatonin and the relative indole compounds on perilla oil oxidation. Korean J. Food Sci. Technol. 48, 610–617. doi: 10.9721/KJFST.2016.48.6.610

[ref34] Kim B. J. Cha G. Y. Kim B. R. Kook Y. H. Kim B. J. (2019). Insights from the genome sequence of Mycobacterium paragordonae, a potential novel live vaccine for preventing mycobacterial infections: the putative role of type VII secretion Systems for an Intracellular Lifestyle within Free-Living Environmental Predators. Front. Microbiol. 10:1524. doi: 10.3389/fmicb.2019.01524, PMID: 31333625 PMC6616192

[ref35] Kim W. Lee E. J. Bae I. H. Myoung K. Kim S. T. Park P. J.. (2020). Lactobacillus plantarum-derived extracellular vesicles induce anti-inflammatory M2 macrophage polarization in vitro. J. Extracell. Vesic. 9:1793514. doi: 10.1080/20013078.2020.1793514, PMID: 32944181 PMC7480564

[ref36] Klein G. (2003). Taxonomy, ecology and antibiotic resistance of enterococci from food and the gastro-intestinal tract. Int. J. Food Microbiol. 88, 123–131. doi: 10.1016/S0168-1605(03)00175-2 14596985

[ref37] Lamont J. R. Wilkins O. Bywater-Ekegärd M. Smith D. L. (2017). From yogurt to yield: potential applications of lactic acid bacteria in plant production. Soil Biol. Biochem. 111, 1–9. doi: 10.1016/j.soilbio.2017.03.015

[ref38] Landemaine L. Da Costa G. Fissier E. Francis C. Morand S. Verbeke J.. (2023). *Staphylococcus epidermidis* isolates from atopic or healthy skin have opposite effect on skin cells: potential implication of the AHR pathway modulation. Front. Immunol. 14:1098160. doi: 10.3389/fimmu.2023.1098160, PMID: 37304256 PMC10250813

[ref39] Lebeer S. Oerlemans E. F. M. Claes I. Henkens T. Delanghe L. Wuyts S.. (2022). Selective targeting of skin pathobionts and inflammation with topically applied lactobacilli. Cell Rep. Med. 3:100521. doi: 10.1016/j.xcrm.2022.100521, PMID: 35243421 PMC8861818

[ref40] Lv J. Sharma A. Zhang T. Wu Y. Ding X. (2018). Pharmacological review on Asiatic acid and its derivatives: a potential compound. SLAS Technol. 23, 111–127. doi: 10.1177/2472630317751840 29361877

[ref41] Majchrzak W. Motyl I. Smigielski K. (2022). Biological and Cosmetical importance of fermented raw materials: an overview. Molecules 27:4845. doi: 10.3390/molecules27154845, PMID: 35956792 PMC9369470

[ref42] Mayslich C. Grange P. A. Dupin N. (2021). Cutibacterium acnes as an opportunistic pathogen: an update of its virulence-associated factors. Microorganisms 9:303. doi: 10.3390/microorganisms9020303, PMID: 33540667 PMC7913060

[ref43] Mazziotta C. Tognon M. Martini F. Torreggiani E. Rotondo J. C. (2023). Probiotics mechanism of action on immune cells and beneficial effects on human health. Cells 12:184. doi: 10.3390/cells12010184, PMID: 36611977 PMC9818925

[ref44] Noda M. Kanno K. Danshiitsoodol N. Higashikawa F. Sugiyama M. (2021). Plant-derived Lactobacillus paracasei IJH-SONE68 improves chronic allergy status: a randomized, double-blind, placebo-controlled clinical trial. Nutrients 13:22. doi: 10.3390/nu13114022, PMID: 34836277 PMC8623948

[ref45] Otto M. (2009). Staphylococcus epidermidis — the ‘accidental’ pathogen. Nat. Rev. Microbiol. 7, 555–567. doi: 10.1038/nrmicro2182, PMID: 19609257 PMC2807625

[ref46] Ou C.-C. Lu T. M. Tsai J. J. Yen J. H. Chen H. W. Lin M. Y. (2009). Antioxidative effect of lactic acid Bacteria: intact cells vs. intracellular extracts. J. Food Drug Anal. 17, 209–216. doi: 10.38212/2224-6614.2609

[ref47] Papadimitriou K. Zoumpopoulou G. Foligne B. Alexandraki V. Kazou M. Pot B.. (2015). Discovering probiotic microorganisms: in vitro, in vivo, genetic and omics approaches. Front. Microbiol. 6:58. doi: 10.3389/fmicb.2015.00058 25741323 PMC4330916

[ref48] Park K. S. (2021). Pharmacological effects of *Centella asiatica* on skin diseases: evidence and possible mechanisms. Evid. Based Complement. Alternat. Med. 2021, 1–8. doi: 10.1155/2021/5462633 PMC8627341 34845411

[ref49] Pieniz S. Andreazza R. Anghinoni T. Camargo F. Brandelli A. (2014a). Probiotic potential, antimicrobial and antioxidant activities of Enterococcus durans strain LAB18s. Food Control 37, 251–256. doi: 10.1016/j.foodcont.2013.09.055

[ref50] Pieniz S. Andreazza R. Okeke B. C. Camargo F. A. O. Brandelli A. (2014b). Assessment of beneficial properties of Enterococcus strains: assessment of beneficial properties of Enterococcus. J. Food Proc. Preserv. 38, 665–675. doi: 10.1111/jfpp.12016

[ref51] Pieniz S. Andreazza R. Okeke B. C. Camargo F. A. Brandelli A. (2015). Antimicrobial and antioxidant activities of Enterococcus species isolated from meat and dairy products. Braz. J. Biol. 75, 923–931. doi: 10.1590/1519-6984.02814 26675908

[ref52] Rafique N. Jan S. Y. Dar A. H. Dash K. K. Sarkar A. Shams R.. (2023). Promising bioactivities of postbiotics: a comprehensive review. J. Agric. Food Res. 14:100708. doi: 10.1016/j.jafr.2023.100708

[ref53] Rashid M. H.-O. Akter M. M. Uddin J. Islam S. Rahman M. Jahan K.. (2023). Antioxidant, cytotoxic, antibacterial and thrombolytic activities of *Centella asiatica* L.: possible role of phenolics and flavonoids. Clin. Phytoscience 9, 1–9. doi: 10.1186/s40816-023-00353-8

[ref55] Sato N. Uematsu M. Fujimoto K. Uematsu S. Imoto S. (2023). Ggkegg: analysis and visualization of KEGG data utilizing the grammar of graphics. Bioinformatics 39:btad622. doi: 10.1093/bioinformatics/btad622, PMID: 37846038 PMC10612400

[ref56] Sen T. Samanta S. K. (2015). Medicinal plants, human health and biodiversity: a broad review. Adv. Biochem. Eng. Biotechnol. 147, 59–110. doi: 10.1007/10_2014_273 25001990

[ref57] Shakya S. Danshiitsoodol N. Noda M. Inoue Y. Sugiyama M. (2022). 3-Phenyllactic acid generated in medicinal plant extracts fermented with plant-derived lactic acid bacteria inhibits the biofilm synthesis of *Aggregatibacter actinomycetemcomitans*. Front. Microbiol. 13:991144. doi: 10.3389/fmicb.2022.991144 36212837 PMC9539679

[ref58] Shakya S. Danshiitsoodol N. Noda M. Sugiyama M. (2023). Role of phenolic acid metabolism in enhancing bioactivity of Mentha extract fermented with plant-derived *Lactobacillus plantarum* SN13T. Probiot. Antim. Prot. 16, 1052–1064. doi: 10.1007/s12602-023-10103-4 PMC11126511 37278953

[ref59] Shokryazdan P. Faseleh Jahromi M. Liang J. B. Ho Y. W. (2017). Probiotics: from isolation to application. J. Am. Coll. Nutr. 36, 666–676. doi: 10.1080/07315724.2017.1337529 28937854

[ref60] Sun B. Wu L. Wu Y. Zhang C. Qin L. Hayashi M.. (2020). Therapeutic potential of Centella asiatica and its triterpenes: a review. Front. Pharmacol. 11:568032. doi: 10.3389/fphar.2020.568032, PMID: 33013406 PMC7498642

[ref61] Tatusov R. L. Fedorova N. D. Jackson J. D. Jacobs A. R. Kiryutin B. Koonin E. V.. (2003). The COG database: an updated version includes eukaryotes. BMC Bioinform. 4:41. doi: 10.1186/1471-2105-4-41, PMID: 12969510 PMC222959

[ref62] Teneva D. Denev P. (2023). Biologically active compounds from probiotic microorganisms and plant extracts used as biopreservatives. Microorganisms 11:1896. doi: 10.3390/microorganisms11081896, PMID: 37630457 PMC10458850

[ref63] van Loo G. Bertrand M. J. M. (2023). Death by TNF: a road to inflammation. Nat. Rev. Immunol. 23, 289–303. doi: 10.1038/s41577-022-00792-3, PMID: 36380021 PMC9665039

[ref64] Yadav M. Mandeep Shukla P. (2020). Probiotics of diverse origin and their therapeutic applications: a review. J. Am. Coll. Nutr. 39, 469–479. doi: 10.1080/07315724.2019.1691957, PMID: 31765283

[ref65] Yu A. O. Leveau J. H. J. Marco M. L. (2020). Abundance, diversity and plant-specific adaptations of plant-associated lactic acid bacteria. Environ. Microbiol. Rep. 12, 16–29. doi: 10.1111/1758-2229.12794, PMID: 31573142

[ref66] Zukiewicz-Sobczak W. Wroblewska P. Adamczuk P. Silny W. (2014). Probiotic lactic acid bacteria and their potential in the prevention and treatment of allergic diseases. Cent. Eur. J. Immunol. 1, 104–108. doi: 10.5114/ceji.2014.42134, PMID: 26155109 PMC4439985

