# Supplementary material for: Isolation, genomic analysis and functional characterization of Enterococcus rotai CMTB-CA6, a putative probiotic strain isolated from a medicinal plant Centella asiatica
Source: Front Microbiol. 2024 Sep 9;15:1452127. doi: 10.3389/fmicb.2024.1452127 (PMC11423741; doi:10.3389/fmicb.2024.1452127)
Supplement: Supplementary file 1 [file Data_Sheet_1.PDF]

*Supplementary Material*

# Isolation, Genomic Analysis, and Functional Characterization of *Enterococcus rotai* CMTB-CA6, a Potential Probiotic Strain from a Medicinal Plant *Centella asiatica*

**Yunsik Kim, Jin Hee Lee, Jimyeong Ha, Eun-Gyung Cho***

*** Correspondence:** Eun-Gyung Cho: egcho@chamc.co.kr

## 1. Supplementary Figure

**A** API 50CHE

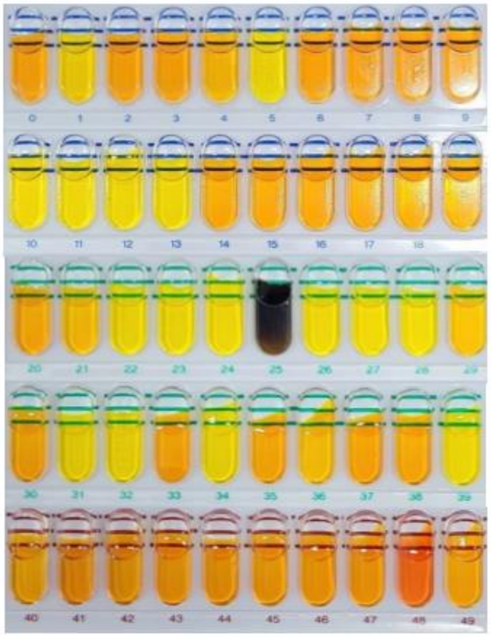

**B**

| API 50CHE characteristic | CMTB-CA6 |
|---|---|
| Glycerol | + |
| Erythritol | - |
| D-Arabinose | - |
| L-Arabinose | - |
| Ribose | + |
| D-Xylose | - |
| L-Xylose | - |
| Adonitol | - |
| B-Methyl-Xyloside | - |
| Galactose | + |
| D-Glucose | + |
| D-Fructose | + |
| D-Mannose | + |
| L-Sorbose | - |
| Rhamnose | - |
| Dulcitol | - |
| Inositol | - |
| Mannitol | - |
| Sorbitol | - |
| α-Methyl-D-Mannose | - |
| α-Methyl-D-Glucoside | - |
| N-Acetyl glucosamine | + |
| Amygdaline | + |
| Arbutine | + |
| Esculine | + |
| Salicine | + |
| Cellobiose | + |
| Maltose | + |
| Lactose | - |
| Melibiose | - |
| Saccharose | + |
| Trehalose | + |
| Inuline | - |
| Melezitose | + |
| D-Raffinose | - |
| Amidon | - |
| Glycogen | - |
| Xylitol | - |
| β-Gentiobiose | + |
| D-Turanose | - |
| D-Lyxose | - |
| D-Tagatose | - |
| D-Fucose | - |
| L-Fucose | - |
| D-Arabitol | - |
| L-Arabitol | - |
| Gluconate | - |
| 2 ceto-gluconate | - |
| 5 ceto-gluconate | - |

**C**

| API 20E characteristic | CMTB-CA6 |
|---|---|
| β-galactosidase | - |
| Arginine dihydrolase | + |
| Lysine decarboxylase | - |
| Ornithine decarboxylase | - |
| Citrate utilization | - |
| $H_2S$ production | - |
| Urease | - |
| Tryptophane desaminase | - |
| Indole production | - |
| Acetoin production | + |
| Gelatinase | - |
| Glucose | + |
| Mannitol | + |
| Inositol | + |
| Sorbitol | + |
| Rhamnose | + |
| Sucrose | + |
| Melibiose | + |
| Arabinose | + |
| Cytochrome oxidase | - |

**Supplementary Figure S1.** Biochemical tests for rapid identification of the CMTB-CA6 strain. Using the analytical profile index (API) 50CHE **(A, B)** and 20E **(C)** systems, the enzymatic activities of the CMTB-CA6 strain were assessed. The reactions occurred under anaerobic conditions, and the results were interpreted after 48 h of incubation. A positive reaction is indicated by "+," whereas a negative reaction is indicated by "–."

2. **Supplementary Table**

**Supplementary Table S1.** The nucleotide identity scores for comparison of the 16S ribosomal RNA (rRNA) gene sequences between CMTB-CA6 and *Enterococcus* spp. strains.

| Rank | Matched bacterial strains | Accession Id | % identity | Total Score | E value |
|---|---|---|---|---|---|
| 1 | *Enterococcus rotai* strain LMG 26678 | CP013655.1 | 99.8 | 2745 | 0 |
| 2 | *Enterococcus rotai* strain CCM 4630 | NR_108137.1 | 99.8 | 2745 | 0 |
| 3 | *Enterococcus* sp. P7644 | AJ276462.1 | 99.8 | 2745 | 0 |
| 4 | *Enterococcus silesiacus* strain LMG 23085 | CP013614.1 | 99.73 | 2739 | 0 |
| 5 | *Enterococcus silesiacus* strain R-23712 | NR_042405.1 | 99.73 | 2739 | 0 |
| 6 | *Enterococcus silesiacus* LMG 23084 | AM039967.1 | 99.67 | 2736 | 0 |
| 7 | *Enterococcus crotali* ETRF1 | NR_156980.1 | 99.93 | 2730 | 0 |
| 8 | *Enterococcus ureilyticus* CCM 4629 | NR_125485.1 | 99.6 | 2728 | 0 |
| 9 | *Enterococcus caccae* 2215-02 | NR_043285.1 | 99.87 | 2724 | 0 |
| 10 | *Enterococcus* sp. HAMBI3059 | FN822762.1 | 99.86 | 2721 | 0 |
